# PD180 Strategies To Minimize The Impact Of The COVID-19 Pandemic On People With Disabilities: Systematic Review And Deliberative Dialogues

**DOI:** 10.1017/S0266462324004057

**Published:** 2025-01-07

**Authors:** Flávia Tavares Elias, Débora Rezende, Maira Ramos

## Abstract

**Introduction:**

The impact of the COVID-19 pandemic was unequal, leading to losses for specific population groups already exposed to social vulnerabilities in the pre-pandemic period—for example, the inequity in access to health care among people with disabilities. The study aimed to identify strategies for people with disabilities during a public health emergency, in particular the COVID-19 pandemic.

**Methods:**

A systematic review was conducted following the PRISMA guidelines. The population was adults with disabilities. There were no restrictions regarding the type of disability, which could include visual, auditory, intellectual, physical, or multiple disabilities. The COVID-19 pandemic was the study exposure and the outcomes were strategies aimed at improving prevention and health care for the target population during the pandemic period. A literature search was conducted in June 2021 and updated in November 2022 in the following databases: PubMed, Web of Science, Scopus, the Virtual Health Library, CINAHL, PDQ-Evidence, Health System Evidence, PEDro, and PsycInfo. The protocol for the systematic review was registered on PROSPERO (CRD42021266341).

**Results:**

The systematic review included 29 studies of 49 non-pharmacological strategies. The evidence was synthesized and structured into categories. The following eight categories were found: habitation and infrastructure; work; occupation and income; planning and management; social assistance; telehealth; communication; comprehensive health care; and education for people with disabilities. The deliberative dialogue allowed stakeholders—represented by people with disabilities, policymakers, decision-makers, health professionals, members of associations, and researchers—to actively engage in constructing the synthesis.

**Conclusions:**

The stakeholder engagement concluded that the project promoted social inclusion and equal, universal, and comprehensive access to social rights by people with disabilities. The experiences of stakeholders in society were incorporated into public policy and guided decision-making in health and social care.

